# Enhancing telehealth services development in Pakistani healthcare sectors through examining various medical service quality characteristics

**DOI:** 10.3389/fpubh.2024.1376534

**Published:** 2024-07-09

**Authors:** Saifullah, Zhiqiang Ma, Mingxing Li, Muhammad Qasim Maqbool, Jing Chen

**Affiliations:** ^1^School of Management, Jiangsu University, Zhenjiang, China; ^2^Department of Management Sciences, University of Okara, Okara, Punjab, Pakistan; ^3^Affiliated Hospital of Jiangsu University, Zhenjiang, China

**Keywords:** medical services quality, telehealth behavior intention, actual use intention, sustainable development, information quality

## Abstract

**Introduction:**

The telehealth service increased attention both during and after the Covid-19 outbreak. Nevertheless, there is a dearth of research in developing countries, including Pakistan. Hence, the objective of this study was to examine telehealth service quality dimensions to promote the telehealth behavior intention and sustainable growth of telehealth in Pakistan.

**Methods:**

This study employed a cross-sectional descriptive design. Data were collected from doctors who were delivering telehealth services through a well-designed questionnaire. To examine the hypothesis of the study, we employed the Smart PLS structural equation modeling program, namely version 0.4.

**Results:**

The study findings indicate that medical service quality, affordability, information quality, waiting time, and safety have a positive impact on the intention to engage in telehealth behavior. Furthermore, the adoption of telehealth behavior has a significant favorable effect on the actual utilization of telehealth services, which in turn has a highly good impact on sustainable development.

**Conclusion:**

The study determined that telehealth services effectively decrease the amount of time and money spent on travel, while still offering convenient access to healthcare. Furthermore, telehealth has the potential to revolutionize payment methods, infrastructure, and staffing in the healthcare industry. Implementing a well-structured telehealth service model can yield beneficial results for a nation and its regulatory efforts in the modern age of technology.

## Introduction

The utilization of telemedicine expanded throughout the COVID-19 pandemic (1). A comprehensive study was conducted in Australia, the United Kingdom, Canada, Italy, Israel, Poland, South Korea, and the United States. They discussed a diverse array of medical concerns. “Among the 14 studies, two were interventions. The participants preferred a mix of in-person visits and telehealth services, but they also showed high satisfaction with video and phone appointments as well as home telemonitoring” (2). In the health sector, telemedicine technology has evolved as a useful tool that assists patients in a variety of ways (3). Nearly all professions, including health, have been affected by Information and Communication Technology (ICT) (4, 5). Telehealth uses “Information Communication Technology (ICT)” to diagnose, consult, and transfer health information to improve patient outcomes (6).

To the benefit, Telehealth makes high-quality healthcare accessible, easy, and motivating for patients (7, 8). Doctors can consult and treat more patients by reducing billable time (9). In addition, Telehealth helps to eliminate deficiencies in healthcare services, cuts down on travel and waiting time, and makes it possible to provide specialist consultations to those living in rural and isolated locations (10, 11). Telehealth is a modern approach that provides fast and specialized healthcare treatments to patients in remote areas with limited access to specialty services. This contributes to sustainable development (12, 13). Besides, Telehealth techniques promote rural “access to healthcare” by improving healthcare operations, quality, and effectiveness (14).

To globally viewpoints, several countries use Telemedicine, such as in America, Southeast Asia, and Europe (15). Its efficacy can be verified in undeveloped regions with few doctors, health facilities, and distances (16). Telemedicine can improve rural healthcare by increasing availability and quality (17). Telemedicine is expanding beyond chronic and common ailments to emergency medical services like ICU, disaster management, and epidemics (18). It reduces transit and indoor crowding, preventing human-to-human transmission in epidemics. Remote screening protects healthy people from hospitals and improves older people adults’ access to care (19). Telemedicine implementation depends on user attitudes. Patient utilization is determined by the patient’s disposition, level of satisfaction with healthcare, and interactions with physicians (20). Besides, secure payment is a major concern in telemedicine (21). Online banking, micro-finance apps, and safe money transfers have made services easier for users and providers (22).

Pakistan spends 38 USD *per capita* on healthcare, substantially less than other developing countries. Compared to Pakistan, India, the Philippines, and Ghana spend 57, 165, and 85 USD *per capita* on healthcare. While Pakistan spent 1.2% of its GDP on public health in 2020–2021, this was not a major rise. The lack of Pakistan health service (PHS) investment has caused a lack of health infrastructure, medicines, medical equipment, and competent healthcare professionals. Human resources increased from 2014 to 2021, but not enough to meet the needs of a 2%-growing population (23). While telehealth services can be beneficial to providing healthcare services to both rural and urban areas, telehealth services also cut travel costs, times, and physical visits.

During pandemic times, Pakistan was facing public hospital’s inadequate infrastructure, dearth of personal protective equipment, lack of isolation rooms and beds, and COVID-19 pandemic-related emergencies led to HCWs’ physical and mental exhaustion, disturbed sleep, mental stress, and fear of infection (24), and while the telemedicine reduced transportation, PPE, and healthcare practitioner effort (25). In this hard time, telemedicine has the potential to reduce the number of hospital visits to prevent transmission, act as a replacement for regular medical consultations, and enhance communication between patients and physicians (26). In the course of the COVID-19 pandemic, telemedicine brought about improvements in healthcare services, saved money, decreased the number of visits to emergency hospitals, and contributed to the spread of the virus (27, 28).

**Figure 1:**
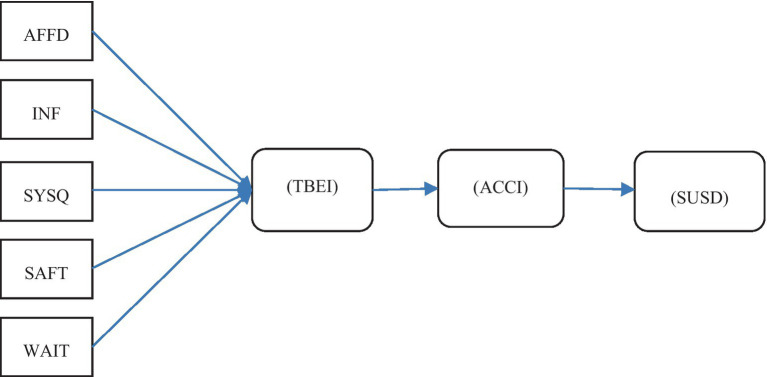
Hypothetical model. AFFD stands for affordability; INF stands for information; SYSQ stands for system quality; WAIT stands for waiting time; SAFT stands for safety; TBEI stands for telehealth behavioral intention; ACC stands for actual use intention; SUSD stands for sustainable development.

Moreover, Pakistan adopted telemedicine in 1988 (29), and in recent times A 2022 systematic review of Pakistani telemedicine noted that the absence of investigations is a significant cause for concern (30). Besides, the legal regulations of telemedicine are currently unavailable in the country (31). The lack of regulatory framework in most countries makes telemedicine integration difficult, yet the COVID-19 pandemic necessitates its adoption (32). To the best of our knowledge, Pakistan lacks a robust Telehealth service quality model. Thus, this study aimed to focus on service quality elements from doctors’ perspectives to create a holistic model. The model of the present study is given in Figure 1. This study confirms that real use affects sustainable development in the telehealth domain which also helps other nations and health services providers to enhance telehealth services and its sustainable development. Therefore this study intends to explore several service dimensions and seeks to answer the following questions:

Q1. How do different service quality dimensions affect telehealth behavioral intention?

Q2. How does telehealth behavioral intention affect actual telehealth utilization in Pakistan?

Q3. How does the practical usage of telehealth affect sustainable development?

## Theoretical background and hypothesis development

The research conducted by Kaium et al. (4) highlighted the appropriateness of the IS System model for examining the persistent intention of both patients and clinicians to utilize Telehealth for consultations. The study suggests that users’ happiness plays a crucial role in their continuous intention to use a particular technology, giving it an advantage over other models of technology adoption (33). Besides, it has been strongly proposed that Information Quality, System Quality, and Medical Service Quality are influential elements in determining both the desire to engage in a certain activity and the actual usage of that behavior (4, 34, 35). Hence, this study regards MSQ as a significant factor that affects the behavioral intention toward Telehealth, subsequently impacting the acceptance of Telehealth among participants.

To Tele-Health and Sustainability. Sustainability refers to the practice of satisfying the current demands while ensuring that the capacity of future generations to fulfill their own needs is not compromised (36). Sustainable development relies on the environment, society, and economy (37). Three must be well-adjusted to avoid overlap. Therefore, it is essential to strategically allocate and govern both organic and human resources in order to optimize financial gain and maintain equilibrium among the three pillars of sustainability, ensuring the well-being of upcoming generations (37). On the other hand, environmental impact specifically targets the reduction of harmful CO2 emissions and water usage throughout the entire energy generation and consumption process (38).

The concept of sustainability is becoming increasingly popular, but, there is a lack of empirical research that has confirmed the sustainability aspect of Tele-Health. Telehealth initiatives possess significant potential to enhance existing healthcare systems in underdeveloped nations. Therefore, this study investigates the relationship between the practical utilization and the advocacy of sustainable development.

### Quality of medical service

Service quality is defined as the user’s evaluation of the services provided by the information system (39). Service quality strongly influences technology adoption intentions (40). Telemedicine adoption failure is connected to low service quality (4). The study outline describes how medical service and information quality affect Telehealth uptake in Pakistan. The change from traditional health checkups to telehealth has implications for both medical professionals and their patients. In addition, the implementation of Telehealth could potentially influence the level of satisfaction among users. This aspect has been extensively discussed in the existing literature on Telehealth, as highlighted by Refs. (6, 41, 42). The Pakistani government and the rural people have faced difficulties in terms of the affordability (AFD) and accessibility of Telehealth services. Possible factors contributing to this issue include the financial constraints faced by local residents, particularly those with limited financial resources, as well as the lack of technological literacy among rural populations. Additionally, inadequate financial support from the government and high fees charged by telehealth service providers may pose challenges in accessing these services. Hence we intended to test the following hypothesis:

*H1*: There is any relationship between affordability service and telehealth behavior intention.

#### Information quality

According to Refs. (43–45), patients could be dissatisfied if the information quality is not pertinent to their health concerns. To increase patient satisfaction, the system’s output must be of the highest quality (41, 46). Preceding research investigations (41, 47–49) have also observed that patient’s ability to easily obtain pertinent information significantly improves patient satisfaction. According to Miller and Sim (49), ensuring adequate maintenance and accessibility of records greatly influences satisfaction with Telehealth services. Similarly, a study on the factors that influence the acceptability of Telehealth in rural Bangladesh. They underlined the importance of accurate information for making informed decisions (50). In Pakistan, Telemedicine is underutilized due to lack of awareness and technology. Telemedicine must be maximized to fix healthcare system flaws. Thus, to maximize telehealth benefits, awareness and infrastructure must be raised (51). In addition to this, the advantageous impact of information that is both current and precise (41). Hence we used the following hypothesis:

*H2*: The quality of the information leads to behavioral intentions toward telehealth behavioral intention.

#### System quality (SYQ)

System functionality, information system usefulness, timeliness, and interoperability are highlighted by DeLone and McLean (39) as the factors that go into system quality. The body of research has demonstrated that SYQ significantly improves users’ happiness with e-health (4, 41). Rahi (52) studied Pakistani Tele-Health users and found that system quality had a major effect on users’ performance. Less effective systems have a detrimental impact on consumers’ intentions to use e-health services (4). However, other investigations refute the assertions supported by other researchers (53) since System Quality was found to be unimportant in the investigation. As a result, it becomes crucial to research how SYQ affects users’ happiness with telehealth since systems must be equipped with sufficient quality features to enable critical functions like diagnosis to be completed without error. Therefore, we proposed the following hypothesis:

*H3*: There is any combination between system quality and telehealth behavioral intention influences.

#### Safety and waiting time

Study (54) noted that medical technology plays an important role in healthcare quality, On the other hand, cleanliness, sanitation, clinician skills, and the capabilities of nurses are considered to be essential characteristics of caring. Telehealth, which is the most recent and cutting-edge technology, makes it simpler to monitor patients who have been released and to manage their rehabilitation, all while saving both the healthcare providers and the patients time and money simultaneously (55). Additionally, it streamlines hospital and clinic workflow. Patients at home who cannot attend the hospital during a pandemic are safer using Tele-Health technology, according to the study. Recently study (56) suggested that the utilization of Telehealth in surgical outpatients is safe and has been verified by evaluations of patient and physician satisfaction, clinical results, and economics. There are some advantages associated with telehealth, such as community access, efficiency, and cost savings; nevertheless, there are also clinical uncertainties, regulatory limits, and safety risks associated with digital safety. Moreover, AlDossary et al. (57) is worth mentioning that Telehealth services are not widely accepted by the majority, lack extensive research that has been reviewed by experts, and exhibit inconsistent levels of depth and quality, which restricts their applicability to a broader context. The study emphasized that Telehealth is driven by technology and characterized by a rapid speed. Therefore, it is recommended to promote the adoption of effective planning and service delivery strategies, enabling others to develop beneficial telehealth programs. Based on the preceding discussion, the subsequent hypothesis has been put forward:

*H4*: There is a correlation between safety and the propensity to engage in telehealth operations.

According to Zhou et al. (41), the main contributors to medical expenses are fees charged by doctors, expenses related to medical check-ups, and the cost of medications. Consequently, improving medical insurance alleviates financial strain, resulting in more cheap healthcare costs (42). Telehealth comfortability (CFT) comprises hassle-free home consultations, easy record access, and timely alerts by frontline health personnel (41, 42). Healthcare professionals’ professionalism (PRF), patient interaction, effective coordination of team members, and seamless and efficient communication contribute to Telehealth MSS. All competent medical equipment-delivered health services add to professionalism. Patient service quality depends on safety (STY). Adequate security and qualified, compassionate healthcare personnel boost patient confidence and user pleasure. Finally, waiting time is the most important MSS component. Previous research showed that prompt diagnosis reduces waiting time and enhances the MSQ (41). Hence we proposed the following hypothesis:

*H5*: There is any relationship between waiting time and telehealth behavior intention.

#### Intentional and actual utilization of telehealth services

Telehealth behavior intention (TBEI) is a major aspect and a predictor of how people are going to utilize Telehealth. TBEI is “the likelihood that a person will use a Tele-Health service” (41). TBEI is an important step in changing any habit, which leads to regular use of any health technology (11). People think of behavioral intention as an important concept that helps to translate into behavior. Before the utilization of any technology, research conducted in the past has demonstrated that behavioral purpose is an essential component (5, 6, 9). People who take TBEI may be more likely to use Telehealth programs. So, the related theory is this:

*H6*: The intention to use telehealth services for behavioral purposes leads to the actual use of these services.

#### Usage as well as advancement of telehealth

Telehealth is seen as a feasible option, indicating its significant contribution to healthcare systems. Telehealth addresses multiple barriers in healthcare, such as minimizing travel time, hence enhancing its effectiveness (58). Telehealth enhances healthcare delivery by altering the conventional in-person interaction between patients and healthcare providers (14, 59, 60). Consequently, this has an impact on hospitals in terms of their financial resources and their ability to generate jobs (14). Telehealth improves the efficiency of healthcare, resulting in cost reduction, better allocation of resources, and higher quality healthcare (14). In addition, the recruitment of local individuals and their subsequent training in the operation of Telehealth equipment facilitates the effective execution and long-term growth of the program. Therefore, it aids in reevaluating the interpretation of UN-SDG 3 and 10. Therefore, the theory that is connected to this is:

*H7*: There is a correlation between the actual utilization and sustainable development of telehealth.

## Methods

Based on the study’s nature, this study utilized a cross-sectional descriptive design. To target the participants, the study included healthcare practitioners who conducted Telehealth consultations in both rural and urban hospitals throughout various regions of Pakistan, namely Sindh, Punjab, and KPK.

### Sampling and data collection

In this study, we employed nonprobability sampling strategies. The non-probability sampling technique was chosen because there was no suitable sampling frame available (61). For data collection, we employed snowball and purposive sampling techniques. Initially, we conducted in-person visits to telehealth service providers in order to gather feedback and collect data. Additionally, we requested contact information from them for any doctors who offer telehealth services. We then reached out to these doctors through phone calls and the WhatsApp application to achieve our study objectives. As a result, one doctor recommended utilizing the “Sehat Kahani Telemedicine System,” and we followed their advice. The Sehat Kahani telemedicine system and mobile health application offer teleconsultation services in Pakistan (62). The purposive sampling was preferred because responders knew little about the occurrence (63). Purposive and snowball sampling were used to collect data from Telehealth practitioners since it was difficult to find a list. Also, prior researchers (64) advised mixed-method sampling because it reduces bias and time. The data were collected from those practitioners who used telehealth. Besides, when the research analysis is carried out, it is necessary to have a sample size of at least 200 observations to develop fit measures that are reliable through the use of structural equation modeling (65, 66). In our study, a total of 525 responses were gathered of which 518 respondents were considered, and the remaining were excluded due to insufficient information. To meet the requirements of the study, two master’s students were employed to gather data from individuals involved in telemedical services. Due to the absence of a comprehensive and officially registered telemedical list, the process of collecting data was challenging and time-consuming, spanning around 4 months from July to October in the year 2023.

#### Survey questionnaire

This study utilized questionnaires obtained from prior researchers. Affordability with four items, information quality, safety system quality, and waiting time with four items from Refs. (41, 67), Actual use intention with three items (11), and Telehealth behavioral intention with three items from Refs. (11, 41). Moreover Sustainable development with three items (14). The evaluation of each item was with a five-point Likert scale strong disagree to strong agree. Before data collection, we involved 20 doctors who had provided telehealth services at a hospital to evaluate the content, phrasing, sequence, design, difficulty, and demands of the questions and the Likert scale. In testing the questionnaire items, 16 doctors suggested that should include an item in telehealth intention about advising others to provide telehealth services and an item that telehealth saves travel cost and time. Hence we add two items one in the telehealth intention that “I will advise others to provide telehealth service to patients” and one into actual use that “Tele-Health is a good idea to save travel cost and time of patients.” After adding these two items questioners were resend to revise. While all the participants were satisfied with the elements and items the questionnaire survey was conducted.

#### Statistical method

Based on the study nature and for validity, we used PLS-SEM (partial least squares structural equation modeling). PLS-SEM has two models: the measurement model, which is also called the outer model, and the inner model, which is also called the structural model. PLS-SEM is now a widely used method for examining intricate connections between observable and latent variables. PLS-SEM is highly valued by researchers due to its several benefits, including the ability to estimate intricate models and its flexibility in terms of data needs and measurement specifications (68). Hence we used this method to test the study hypothesis and model. According to scholars Smart PLSM4 (68) bootstrapped data to determine loadings, weights, and path coefficient significance levels. As advised by Hair et al. (69), initially, we assess the validity and reliability of the measurement model, followed by the examination of the structural linkages in the structural model. The Smart PLS program version 0.4 was employed for data analysis and hypothesis testing in the discipline of statistical analysis.

## Results

### Personal information

This study investigated the sustainable development of telehealth by examining the participation of medical practitioners who have given telehealth services. Demographical information is given in Table 1. The study was done in the rising nation of Pakistan. 43.5% of the respondents were women and 56.9% were male. The respondents possessed varying qualifications, with 48.4% holding an MBBS degree, 38.03% holding an MD degree, and 8.8% holding a bachelor in ayurvedic medicine and surgery BAMS degree. Only 4.6% had qualifications other than these. The respondents were associated with various job types, with 36.6% working in the government sector, 59.6% in the private sector, and 3.6% in private practice. In addition to Tele-consultation, the distribution of monthly patient volumes is as follows: 27.7% of doctors treat less than five patients, 52.7% treat between 5 and 10 patients, 13.7% treat between 10 and 15 patients, and only 5.7% treat more than 15 patients in a month. In the past 6 months, a small number of patients were treated with varying percentages: less than 10 patients were treated with 30.30%, 10–20 patients were treated with 26.06%, 20–30 patients were treated with 36.6%, and more than 30 patients were treated with 6.9% (Table 1).

**Table 1:** Personal information (*N* = 518).

	Frequency	Percentage %
Gender		
Female	223	43.05
Male	295	56.94
Education		
MBBS	251	48.45
MD	197	38.03
BAMS	46	8.88
Other	24	4.63
Type of Job		
Government	190	36.67
Private	309	59.65
Private Practice	19	3.66
Monthly Tele-consultation		
<5	144	27.79
5–10	273	52.70
10–15	71	13.70
Above 15	30	5.79
In the last 6 months, patients were treated with telemedicine		
<10	157	30.30
10–20	135	26.06
20–30	190	36.67
Above 30	36	6.94

#### Construct reliability and validity

All of the CR, AVE, and Outer Loadings values are found to be appropriate, as seen in Table 2, and convergent validity has been established. Actual Use Intention’s Alpha (0.812), reliability (0.872) and average (0.630), Affordability’s Alpha (0.802), reliability (0.868) and average (0.621), Information’s Alpha (0.773), reliability (0.842) and average (0.574), Safety’s Alpha (0.857), reliability (0.912) and average (0.777), Sustainable Development’s Alpha (0.599), reliability (0.787) and average (0.569), System Quality’s Alpha (0.915), reliability (0.947) and average (0.885), and Tele-Health Behavior Intention’s Alpha (0.781), reliability (0.861) and average (0.612) and Waiting Time’s Alpha (0.941), reliability (0.962) and average (0.894). In accordance with the findings of Hair et al. (70), convergent validity is established whenever the CR, AVE, and outer loadings are more than 0.50, 0.70, and 0.60, respectively. An evaluation of the degree of consistency that exists between various indicators of the same construct is what is meant by the term “convergent validity.” When the values of AVE, CR, and outer loadings are between 0 and 1, it constitutes the establishment of the condition. Additionally, in order to guarantee convergent validity, the AVE value absolutely must be greater than 0.50 (69). According to the authors of the study, all of the AVEs in our investigation are valid above 0.50, indicating promising outcomes. The validity of the content is authenticated by the factor loading of several different items. Through the use of factor loading, it is ensured that the construct questions will measure what they are intended to test and that they are pertinent to the particular construct being tested (71). It is imperative that the factor loading not be lower than 0.7. The results of our study are trustworthy.

**Table 2:** Construct reliability and validity.

Items	F. Loadings	*C. alpha*	C. reliability (rho_c)	AVE	VIF
Actual Use Intention					
ACCI1	0.756	0.812	0.872	0.630	1.599
ACCI2	0.824				2.520
ACCI3	0.794				2.451
ACCI4	0.799				1.366
Affordability					
AFD1	0.752	0.802	0.868	0.621	1.648
AFD2	0.774				1.808
AFD3	0.799				1.420
AFD4	0.826				1.821
Information					
INF1	0.816	0.773	0.842	0.574	1.816
INF2	0.822				1.318
INF3	0.775				1.605
INF4	0.706				1.499
Safety					
SAFE1	0.750	0.857	0.912	0.777	1.576
SAFE2	0.945				3.602
SAFE3	0.936				3.536
Sustainable Development					
SUSD1	0.835	0.599	0.787	0.569	1.530
SUSD2	0.762				1.077
SUSD3	0.892				1.614
System Quality					
SYSQ1	0.922	0.915	0.947	0.855	3.029
SYSQ2	0.935				3.581
SYSQ3	0.917				3.123
Tele-Health Behavior Intention					
TBEI1	0.719	0.781	0.861	0.612	1.132
TBEI2	0.836				2.133
TBEI3	0.830				2.083
TBEI4	0.822				1.937
Waiting Time					
WAIT1	0.947	0.941	0.962	0.894	4.452
WAIT2	0.946				4.432
WAIT3	0.943				4.127

#### Discriminant validity (HTMT)

HTMT correlation ratio measures discriminant validity. The HTMT correlation, created by Henseler and Sarstedt (72), is a powerful discriminant validity tool. To prove validity, the HTMT value should be less than 0.90 (73). HTMT values over 0.90 indicate discriminant validity issues. Table 3 reveals that all constructions had HTMT ratios below 0.90. AFD (0.793), INF (0.765), SAFE (0.394), SUSD (0.808), and SYSQ (0.605), TBEI (0.742) and WAIT (0.532). This suggests the constructs have sufficient validity and the model is worthy.

**Table 3:** Discriminant validity (HTMT).

Items	ACCI	AFD	INF	SAFE	SUSD	SYSQ	TBEI	WAIT
ACCI								
AFD	0.793							
INF	0.765	0.735						
SAFE	0.394	0.317	0.359					
SUSD	0.808	0.746	0.802	0.835				
SYSQ	0.605	0.640	0.702	0.363	0.866			
TBEI	0.742	0.693	0.757	0.454	0.784	0.777		
WAIT	0.532	0.559	0.689	0.291	0.783	0.767	0.746	

#### Discriminant validity – Fornell-Larcker criterion

The model’s latent variables’ shared variance is commonly quantified using criteria (74). This criterion can assess the measurement model’s convergent validity using AVE and CR. Fornell-Larcker and cross-loading measure discriminant validity. Table 4 indicates that each construct’s off-diagonal values are smaller than AVE square roots, meeting the Fornell-Larcker requirement valid.

**Table 4:** Discriminate validity Fornell-Larcker criterion.

Items	ACCI	AFD	INF	SAFE	SUSD	SYSQ	TBEI	WAIT
ACCI	0.793							
AFD	0.640	0.788						
INF	0.627	0.598	0.757					
SAFE	0.356	0.288	0.325	0.881				
SUSD	0.619	0.586	0.702	0.503	0.754			
SYSQ	0.562	0.577	0.646	0.329	0.762	0.825		
TBEI	0.631	0.576	0.681	0.386	0.704	0.660	0.782	
WAIT	0.500	0.522	0.645	0.252	0.642	0.711	0.644	0.946

#### Coefficient of determination

Consider the effect size and variation generated by independent variables, as well as the model’s prediction accuracy as assessed by R^2^ are given in Table 5. The MSQ accounts for 59% of TBEI variance, according to the R^2^ of 0.596. The combined variance of TBEI and AACI is 0.39, hence the TBEI R^2^ value changes ACCI by 39.8%. SUSD R^2^ is 0.38, indicating that ACCI causes 0.38% of SUSD (Table 5). It shows that medical service quality is closely linked to telehealth development, behavior, and use.

**Table 5:** Coefficient of determination (R^2^).

	R-square	R-square adjusted
ACCI	0.398	0.397
SUSD	0.383	0.382
TBEI	0.596	0.592

#### Testing hypothesis and structural model

**Figure 2:**
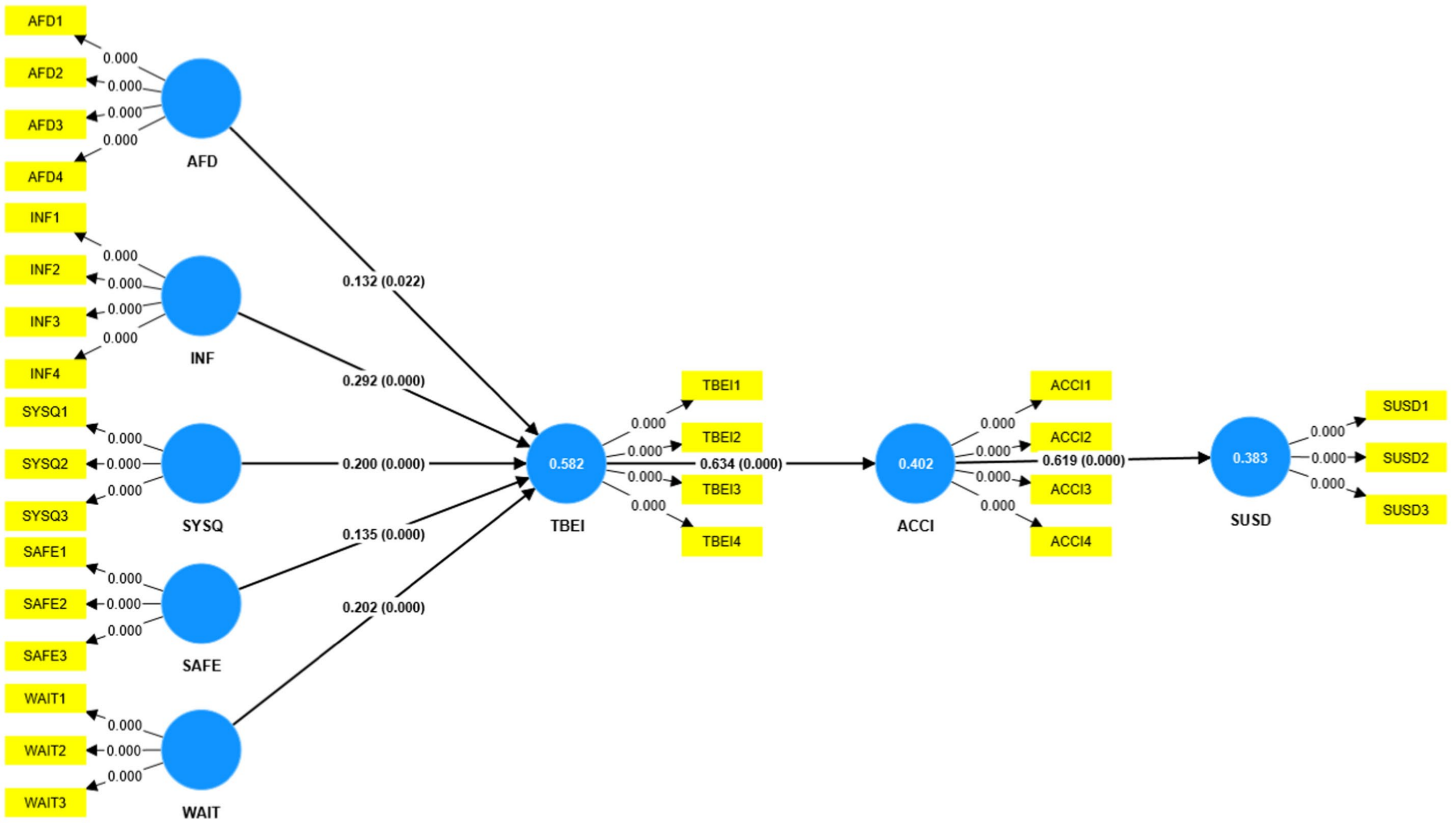
Structure equation model (Boat Strapping).

The structural model illustrates the proposed correlation between the constructs of the paradigm. The coefficients in PLS-SEM modeling are equivalent to the coefficients obtained in ordinary regression analysis. The coefficient measures the extent to which the independent variable causes variation in the dependent variable. The beta coefficient also signifies the statistical importance of the proposition. A high beta value signifies a substantial impact on an independent variable. However, the importance of the link can only be confirmed by the coefficient value, although T-statistics can aid in assessing its significance. To determine the importance of a hypothesis, the Bootstrapping technique was employed to compute significant values (75). The structure model of the testing hypothesis is shown in Figure 2.

The results of the bootstrapping are given in Table 6. We examined the MSQ’s five dimensions’ effects on TBEI, ACCI, and sustainable development (SUSD). We predicted MSQ would boost TBEI significantly. Table 6 confirms that all study hypotheses are supported and accepted. H1 predicted a significant favorable effect of AFFD on TBEI (*β* = 0.131, T-stat = 2.290, *p* > 0.022). Therefore, hypothesis 1 is substantially supported by the facts. Our investigation found a substantial favorable impact of INF on TBEI (*β* = 0.299, T-stat = 5.578, *p* > 0.000). Thus, H2 is supported in this investigation. In H3, we projected that system quality (SYSQ) would boost TBEI significantly. The results (*β* = 0.199, T-stat = 4.224, *p* > 0.000) reveal a positive and substantial link between system quality and Telehealth behavior intention. Our study found that safety (SAFE) affects telehealth behavior intention (TBEI) (*β* = 0.134, T-stat = 3.521, *p* > 0.000). Support and acceptance of H4. WAIT was expected to be favorable and significant to Telehealth behavior intention (TBEI) for H5. The significance has a favorable influence (*β* = 0.197, T-stat = 3.622, *p* > 0.000).

**Table 6:** Hypothesis testing (Bootstrapping).

Hypothesis Description		Path Coefficients	Standard deviation	T statistics	*p*-values	Conclusion
H1.	AFD - > TBEI	0.131	0.058	2.290	0.022	Supported
H2.	INF - > TBEI	0.299	0.052	5.578	0.000	Supported
H3.	SYSQ - > TBEI	0.199	0.047	4.224	0.000	Supported
H4.	SAFE - > TBEI	0.134	0.038	3.521	0.000	Supported
H5.	WAIT - > TBEI	0.197	0.056	3.622	0.000	Supported
H6.	TBEI - > ACCI	0.637	0.026	24.017	0.000	Supported
H7.	ACCI - > SUSD	0.621	0.026	24.261	0.000	Supported

The significance level is *p* < 0.001 and *p* < 0.05.

In addition, H6 anticipated that Telehealth behavior intention positively affects actual use intention. The study found a positive association between TBEI and ACCI (*β* = 0.637, T-stat = 24.017, *p* > 0.000). Our last hypothesis examined actual (ACCI) use and sustainable development (“SUSD”). The study indicates a positive association between ACCI and SUSD (*β* = 0.621, T-stat = 24.261, *p* > 0.000). The study shows all hypotheses are true. This study helps emerging nations implement telehealth services that could be advantageous if medical service quality is valid.

## Discussion

This study predicts MSQ (Medical Service Quality) and assesses its influence on individuals’ intention to engage in Telehealth services. This study investigates the influence of information and system quality on engaging in Telehealth activity. The study investigates the correlation between the practical application of Telehealth and the goal to promote sustainable development in Pakistan. Telemedicine adoption failure is connected to low service quality (4). Besides, it has been strongly proposed that Information Quality, System Quality, and Medical Service Quality are influential elements in determining both the desire to engage in a certain activity and the actual usage of that behavior (4, 34, 35).

Safety and waiting time were loaded higher than comfortability, professionalism, and cost in the proposed conceptual framework for MSQ. These findings support prior research indicating safety issues may decrease practitioner and patient confidence (76). This suggests practitioners worry about the online storage of sensitive data. Next in importance was waiting time. Telehealth also saves time, according to previous studies (77, 78). Telehealth has solved travel, parking, and practitioner waiting rooms. Telehealth comfortability (CFT) comprises hassle-free home consultations, easy record access, and timely alerts by frontline health personnel (41, 42). Moreover, waiting time is the most important MSS component. Previous research showed that prompt diagnosis reduces waiting time and enhances the MSQ (41).

According to previous studies (6, 41), MSQ was found to have a significant and positive impact on traumatic brain injury (TBI). The telehealth programs and quality services provided at telehealth facilities with appropriate diagnosis tend to improve traumatic brain injury (TBI), reduce the amount of money spent on medical care, and additionally enhance practitioners’ satisfaction (79). According to Zhou et al. (41), the main contributors to medical expenses are fees charged by doctors, expenses related to medical check-ups, and the cost of medications.

The study also confirmed that information quality affects TBEI, based on previous studies (41, 46, 79). The outcomes showed that INQ had a greater impact than MSQ and SYQ, highlighting the importance of early and accurate information in diagnosis and therapy. According to Refs. (45–47), patients may be unsatisfied with unrelated health information. And easy access to relevant information boosts patient satisfaction (43, 49–51). Poor systems reduce users’ e-health service intents (6), and other studies disprove the claims of other researchers (55) because System Quality is unimportant. Digital possession of health data lets doctors provide value-based care. Previous research implies that correct and therapeutically relevant information aids diagnosis (80). Telehealth behavioral intention positively impacted actual use, consistent with other studies showing a high TBEI effect on actual use. TBEI is “the likelihood that a person will use a Tele-Health service” (41). TBEI is an important step in changing any habit, which leads to regular use of any health technology (11).

This study’s main contribution is that sustainable development is linked with actual use and effective service quality expanding the approach to sustainable development. Telehealth addresses multiple barriers in healthcare, such as minimizing travel time, hence enhancing its effectiveness (58). Previous research has shown that emphasizing Telehealth could help all involved parties (patients, attendants, healthcare professionals) and contribute to business sustainability. “Moving hospital to patients” could contribute to sustainable development by providing high-value healthcare in underdeveloped or distant places. Focusing on Tele-Health can implement patient-centric “smooth treatments which include evaluation, follow-up including specialized healthcare consultations. Telehealth enhances healthcare delivery by altering the conventional in-person interaction between patients and healthcare providers (14, 59, 60).

## Conclusion

To gain a better knowledge of the relative significance of MSQ, INQ, and SYS in the use of Telehealth, this study explored the role that each of these factors plays in the process. This study provides more data to support the influence of real-world applications for sustainable growth. As a result, the study confirms the relationships between causes and effects and establishes a comprehensive hypothesis which in turn enhances the usefulness of the constructions within the context of a developing nation. According to the findings of the study, telehealth is having a huge impact on healthcare all over the world and is bringing about a technological revolution in the field of medical sciences. The participants demonstrated a respectable level of skill in the delivery of telemedical services and indicated a positive view regarding the numerous components and benefits of telemedicine in Pakistan. Telemedicine has made it possible to overcome geographical and temporal limitations, as well as increased the availability of high-quality medical care, reduced the costs associated with medical treatment, and reduced the overall cost of medical care. Telemedicine can help alleviate the shortage of resources as well as the insufficient number of doctors allocated to each patient. In addition, there is a connection between service provision and sustainable development in telehealth. Positive progress can be made in the direction of telehealth if the telehealth industry adopts a good framework.

## Limitations and potential future directions

This study has several limitations. First, the study was conducted in Pakistan which can be extended to other developing countries through using different variables and dimensions. Second, the study covers doctors’ perspectives, allowing researchers to reproduce it with patients and compare results. Third, Telehealth may also enhance patient satisfaction. Fourth, the investigation could confirm these traits for rural settings at national and regional levels. Further research may validate legislation, regulatory structures, financial issues, and hurdles to creating a structure at the national and regional levels. Finally, the researcher may face challenges in the data collecting process in the study content.

## Data availability statement

The original contributions presented in the study are included in the article/Supplementary material, further inquiries can be directed to the corresponding authors.

## Ethics statement

The studies involving human participants were reviewed and approved by the ethics committee at Jiangsu University China. The patients/participants provided their written informed consent to participants in this study. The studies were conducted in accordance with the local legislation and institutional requirements. Written informed consent for participation in this study was provided by the participants’ legal guardians/next of kin.

## Author contributions

Saifullah: Writing – review & editing, Writing – original draft. ZM: Writing – review & editing, Supervision, Funding acquisition. ML: Writing – review & editing, Project administration, Funding acquisition. MM: Writing – review & editing, Software, Methodology, Investigation, Formal analysis, Data curation. JC: Writing – review & editing.
