# Supplementary material for: Enhancing telehealth services development in Pakistani healthcare sectors through examining various medical service quality characteristics
Source: Front Public Health. 2024 Jul 9;12:1376534. doi: 10.3389/fpubh.2024.1376534 (PMC11263101; doi:10.3389/fpubh.2024.1376534)
Supplement: Supplementary file 1 [file Table_1.docx]

**Survey Questioner Information:**

**Section-I: Personal Information:**

| **Note:** Only telehealth providers are required to complete the following questionnaire. Please provide your response within the designated quotation marks. We appreciate your time and help. | | | | |
| --- | --- | --- | --- | --- |
| Gender | - Male | - Female | - Other |  |
| Education | - MBBS | - MD | - BAMS | - Other |
| Type of Job | - Government | - Private | - Private Practice | - Other |
| Tele-consultation in month | - Less than 5 | - 5-10 | - 10-15 | - 15-20 |
| In last 6 month patient treated through telemedince | - 5-10 | - 10-20 | - 20-30 | - Above 30 |

**Section-II: Questions**

| **Note: Please rate the responses of each question with a tick mark.** | | | | |
| --- | --- | --- | --- | --- |
| - 1.Strongly Disagree | - 2.Disagree | - 3.Natural | - 4. Agree | - 5. Strongly Agree |

| **Affordability** | | | | | |
| --- | --- | --- | --- | --- | --- |
| The total cost of the Tele-Health consultation is affordable for patients. | - 1 | - 2 | - 3 | - 4 | - 5 |
| I take a reasonable fee from rural patients for Tele-Consultation. | - 1 | - 2 | - 3 | - 4 | - 5 |
| Drug and other equipment charges are reasonable. | - 1 | - 2 | - 3 | - 4 | - 5 |
| Cost of medical examination is clear. | - 1 | - 2 | - 3 | - 4 | - 5 |
| **Information quality** | | | | | |
| Information provided by nurses regarding the patients is apt. | - 1 | - 2 | - 3 | - 4 | - 5 |
| Tele-Health can provide useful information about common disease prevention. | - 1 | - 2 | - 3 | - 4 | - 5 |
| The information provided about first aid measures in correct. | - 1 | - 2 | - 3 | - 4 | - 5 |
| The information provided through Tele-Health helps me to provide adequate medication. | - 1 | - 2 | - 3 | - 4 | - 5 |
| **System Quality** | | | | | |
| Tele-Health is reliable source of connecting with patients at remote centers. | - 1 | - 2 | - 3 | - 4 | - 5 |
| The ergonomic design of Tele-Consultation increases my productivity | - 1 | - 2 | - 3 | - 4 | - 5 |
| I can afford to provide Tele-Consultation due to peripheral devices | - 1 | - 2 | - 3 | - 4 | - 5 |
| **Safety** | | | | | |
| The treatment provided by through Tele-Consultations is safe. | - 1 | - 2 | - 3 | - 4 | - 5 |
| I can handle complicated cases through Tele-Consultations. | - 1 | - 2 | - 3 | - 4 | - 5 |
| The drugs suggested by me through Tele-Consultation are safe. | - 1 | - 2 | - 3 | - 4 | - 5 |
| **Waiting Time** | | | | | |
| The waiting time for patients to join online is tolerable. | - 1 | - 2 | - 3 | - 4 | - 5 |
| The waiting time for providing diagnosis is tolerable. | - 1 | - 2 | - 3 | - 4 | - 5 |
| The waiting time for nurse to translate in case of vernacular language is tolerable. | - 1 | - 2 | - 3 | - 4 | - 5 |
| **Telehealth behavior intention** | | | | | |
| I will provide Tele-Health consultations in the future | - 1 | - 2 | - 3 | - 4 | - 5 |
| I will continue advising patients through Tele-Consultations | - 1 | - 2 | - 3 | - 4 | - 5 |
| I will consider Tele-Health as the first choice | - 1 | - 2 | - 3 | - 4 | - 5 |
| I will advise others to provide telehealth service to patients | - 1 | - 2 | - 3 | - 4 | - 5 |
| **Actual use** | | | | | |
| I am comfortable providing Tele-Consultations | - 1 | - 2 | - 3 | - 4 | - 5 |
| I use Tele-Health as it improves the convenience of medical services | - 1 | - 2 | - 3 | - 4 | - 5 |
| Tele-Health is a good idea to provide medical assistance to patients | - 1 | - 2 | - 3 | - 4 | - 5 |
| Tele-Health is a good idea to save travel cost and time of patients | - 1 | - 2 | - 3 | - 4 | - 5 |
| **Sustainable Development** | | | | | |
| Tele-Health tends to develop a competent workforce. | - 1 | - 2 | - 3 | - 4 | - 5 |
| Tele-Health improves digital ecosystem and develops a national IT infrastructure to support Tele-Health. | - 1 | - 2 | - 3 | - 4 | - 5 |
| Including Tele-Health into routine care leads to sustainable development. | - 1 | - 2 | - 3 | - 4 | - 5 |
